# Transient Neonatal Diabetes Mellitus in a Turkish Patient with Three Novel Homozygous Variants in the ZFP57 Gene

**DOI:** 10.4274/Jcrpe.928

**Published:** 2013-05-30

**Authors:** Mehmet Boyraz, Korkut Ulucan, Necati Taşkın, Teoman Akçay, Sarah E. Flanagan, Deborah J.G. Mackay

**Affiliations:** 1 Şişli Etfal Education and Research Hospital, Division of Pediatric Endocrinology, İstanbul, Turkey; 2 Üsküdar University, Faculty of Engineering and Natural Sciences, Department of Molecular Biology and Genetics, İstanbul, Turkey; 3 Kanuni Sultan Süleyman Education and Research Hospital, Division of Pediatrics, İstanbul, Turkey; 4 Sadi Konuk Education of Research Hospital, Division of Pediatric Endocrinology, İstanbul, Turkey; 5 Peninsula College of Medicine and Dentistry, Division of Molecular Genetics, Exeter, UK; 6 University of Southampton, School of Medicine, Human Genetics Research Division, Salisbury, UK

**Keywords:** ZFP57 gene, transient neonatal diabetes mellitus, Turkish, novel mutations

## Abstract

Neonatal diabetes mellitus (NDM) is a rare form of diabetes that presents within the first six months of life. Nearly 70% of these cases have loss of methylation at the differentially methylated region on chromosome 6q24. To describe the findings in a Turkish male patient with NDM caused by a loss of methylation at chromosome 6q24 and three novel homozygous mutations in the ZFP57 gene, methylation-specific PCR was carried out at 6q24 and mutation analysis of ZFP57 gene was maintained by direct sequencing. Sequencing of ZFP57 gene revealed the hypomethylation of chromosome 6q24 and three novel mutations (chr6:29.641.413 A>T, 29.641.073 C>T, and 29.640.855 G>C), respectively. The latter mutation seems to display the patient’s condition due to a highly conservative amino acid substitution in the protein. We suggest the ZFP57 gene as a causative factor for NDM and it should be considered in genetic testing. Further studies including functional analysis of the detected mutations will provide precise information regarding the effect of the mutations.

**Conflict of interest:**None declared.

## INTRODUCTION

Neonatal diabetes mellitus (NDM) is a rare form of diabetes with an incidence of 1/90000 live births. It is defined as diabetes that presents within the first six months of life, persists for at least two weeks, and requires insulin treatment (1). The outcome is highly variable with approximately 40-50% of patients with permanent NDM (PNDM) and 50-60% with transient NDM (TNDM). TNDM resolves postnatally, but may relapse in later life (2,3). Approximately 70% of TNDM cases have loss of methylation at the differentially methylated region (DMR) on chromosome 6q24. Loss of methylation can result from one of three genetic mechanisms: 1) paternal uniparental disomy 2) paternal duplication, or 3) loss of methylation without a structural chromosome defect. The critical region on chromosome 6q24 encompasses PLAGL1, a tumor suppressor gene, and HYMAI, a non-coding RNA of unknown function. The mechanism(s) by which loss of methylation, and hence overexpression of PLAGL1 and/or HYMAI cause TNDM is not known (4,5). Approximately 50% of TNDM patients with a loss of methylation and no structural chromosome defect at chromosome 6q24 have hypomethylation of imprinted loci (HIL) throughout the genome which in many cases results in a syndromic phenotype (6). In a proportion of these patients, recessive mutations in ZFP57 gene are identified. ZFP57 encodes a zinc-finger transcription factor expressed in early development, which is thought to be involved in DNA methylation during the earliest multicellular stages at multiple imprinting control regions (6).

Here, we describe a Turkish patient with TNDM caused by a loss of methylation at the chromosome 6q24 locus and three novel homozygous missense variants in the ZFP57 gene.

**Case Presentation**

A 12-day-old male was referred from another clinic due to feeding difficulties, lethargy, and hyperglycemia. The patient was born to healthy first-degree cousins at 36 weeks’ gestation by cesarean section delivery with a birth weight of 2040 g and birth length of 45 cm (both <3rd percentile). Physical examination did not reveal any dysmorphic features. His weight was 2050 g at admission. He was not receiving parenteral nutrition, and sepsis markers were negative. Hyperglycemia persisted without metabolic acidosis or ketonuria. Laboratory investigations revealed a serum C-peptide of 0.1 nmol/L (normal range, 0.29-1.32 nmol/L), a serum insulin level of 48.2 pmol/L (normal range, 18-186 pmol/L) and a simultaneous blood glucose level of 28 nmol/L. HbA1c was 2.7% (normal range, 2.1-7.7%). There was no evidence of autoimmunity (negative insulin antibodies, glutamic acid decarboxylase antibodies, and islet cell antibodies). Abdominal ultrasonography demonstrated a normal pancreas anatomy. Function studies of other endocrine glands, such as the thyroid and adrenal glands, also revealed normal results. The patient’s brother, who is currently 10 years of age, was diagnosed with NDM, which remitted at the age of 15 days. Neither of the patient’s parents had symptoms of diabetes. The parents’ and brother’s fasting blood glucose levels were within normal limits.

The patient was started on continuous intravenous rapid acting insulin therapy (0.5 U/kg/day) with strict blood glucose monitoring by bedside capillary glucose testing. A high caloric intake was maintained, and a satisfactory weight gain was accomplished. On the 18th day of life, insulin therapy was changed from intravenous infusion to subcutaneous neutral protamine Hagedorn (NPH), with progressive metabolic control. Hypoglycemia developed several times during insulin therapy. The patient was discharged after resolution of hyperglycemia. He remained on NPH insulin treatment until 6 months of age.

At the age of 6 months, and following the genetic diagnosis, the patient entered remission at which stage insulin treatment was withdrawn. The patient is currently 12 months of age. His growth and physical development are normal.

**Molecular Studies**

Genetic profiles were programmed for outpatient follow-up. Genomic DNA was extracted from the peripheral blood of the patient and family members using the High Pure PCR Template Preparation kit (Roche Diagnostics, Mannheim, Germany). The parents provided their written informed consent, and the study was conducted in compliance with the declaration of Helsinki (2000). ABCC8, KCNJ11, INS, and EIF2AK3 genes were sequenced, and no mutations were detected. In addition to these, methylation-specific PCR of the patient’s DNA showed complete loss of maternal methylation at the PLAGL1 DMR on chromosome 6q24, confirming a diagnosis of TNDM due to 6q24 hypomethylation.

DNA methylation was analyzed at other known imprinted regions, using methods as described (7). In addition to the PLAGL1 DMR, DNA hypomethylation was detected at the GRB10, PEG3, and NESP-AS regions, but not at the MEST, ICR1, ICR2, 14q32, or SNRPN regions. This pattern of hypomethylation has been previously observed in individuals with TNDM and mutation of the ZFP57 gene (7).

**Figure 1:**
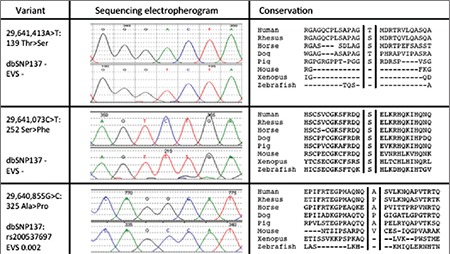
Variants ofZFP57gene (first column), electropherogramresults of sequencing (middle column) and amino acid conservation(left column)

Coding regions of ZFP57, with intron-exon boundaries, were sequenced in the patient and his parents. The patient had three novel homozygous coding variants, p.T139S (chr6:29,641,413A>T), p.S252F (chr6:29,641,073C>T), and p.A325P (chr6:29,640,855G>C) (Figure 1), and all three were found in heterozygous form in the parents.

These results were consistent with a diagnosis of TNDM-HIL resulting from homozygous variation/s of ZFP57. Both parents were heterozygous for these changes. While p.T139S and p.S252F were not present in dbSNP137 (http://www.ncbi.nlm.nih.gov/SNP/) or the Exome Variant Server (http://evs.gs.washington.edu/EVS/), p.A325P was present in dbSNP137 as rs200537697 with a minor allele frequency of 0.15%. p.T139S and p.A325P were changes found within other placental mammals (Figure 1) and were located within relatively weakly conserved stretches of amino acids. By contrast, p.S252F was present in the highly-conserved zinc finger region responsible for DNA binding (8) and, moreover, near to other known variants associated with TNDM-HIL (7,9). Therefore, it seems likely that p.S252F is the variant responsible for the patient’s condition.

## DISCUSSION

NDM is defined as hyperglycemia occurring in the first 6 months of life (10,11). The understanding of the molecular basis for NDM has advanced in recent years. Clinically, this condition can be divided into two categories (TNDM and PNDM), which differ from each other in the resolution or persistence by 18 months of age. However, relapse in childhood or adolescence occurs in up to 50% of cases. Changes in insulin requirements and changes in the number of pancreatic beta cells have been implicated as the cause of this biphasic course, while the exact mechanism remains undetermined (10).

Our patient had been receiving NPH insulin injections for glycemic control before the mutation analysis which excluded ABCC8, KCNJ11, INS, and EIF2AK3 mutations and demonstrated the presence of a methylation abnormality on 6q24. Previous reports have indicated that oral sulfonylurea therapy in patients with mutations of the KATP channel genes, ABCC8 and KCNJ11, can result in glycemic control that is as good, or better, than that achieved with subcutaneous insulin injection (11,12,13,14). Based on the analysis result of our patient, treatment with a sulfonylurea was not initiated as there was already sufficient control with insulin therapy.

ZFP57 gene encodes a zinc-finger protein. Recent studies indicate that ZFP57 is a regulator of transcription required for maintenance of imprinting (15,16), while in humans, this gene has been associated with TNDM (7,9). Novel variation at position 3519 A>T, a transversion change within the gene, leads to the substitution of amino acid threonine to the similarly polar and uncharged serine at position 139 (T139S), while a G>C transversion at position 4077 causes substitution of alanine by the similarly non-polar proline residue (A325P). Both these variations are in relatively non-conserved stretches of amino acids, and the second of them is a recognized though rare SNP. A third novel variation, 3859 A>T, substituted amino acid serine to phenylalanine (S252F). X-ray crystallography studies predict that this substitution is in the highly conserved DNA-binding zinc-finger region of ZFP57, and therefore has an impact on its DNA binding. By the sequencing of gene, we showed the three novel variations in the patient, but we are still far from identifying the effect of these variants on the function of ZFP57 and therefore its physiological effects on our patient.

In conclusion, ZFP57 gene is an important genetic determinant for the onset of TNDM. Considering testing for this gene in diagnosed cases will help clinicians to have more knowledge regarding the effect of variations detected in the gene sequence on the disease. It is difficult to comment on the effect of these changes we detected in this report. Further studies, including analyses of the same gene of the patients’ brother and functional analysis of the gene will help us have more precise information on these newly detected changes.
